# Progressive Spastic Paraparesis as the Dominant Manifestation of Adolescent-Onset Alexander Disease: Case Report and Literature Review

**DOI:** 10.3390/jcm14228232

**Published:** 2025-11-20

**Authors:** Katarzyna Anna Smółka, Leon Smółka, Wiesław Guz, Emilia Chaber, Lidia Perenc

**Affiliations:** 1 Department of Child Neurology and Pediatrics, Clinical Regional Hospital No. 2, 35-301 Rzeszów, Poland; chaberemilia@gmail.com (E.C.); la.perenc@gmail.com (L.P.); 2 Centrum Medyczne Dr Smółka, 32-500 Chrzanów, Poland; 3 Department of Anatomy, Medical University of Silesia, 40-055 Katowice, Poland; s81350@365.sum.edu.pl; 4 Department of Radiology and Imaging Diagnostics, Clinical Regional Hospital No. 2, 35-301 Rzeszów, Poland; wguz@ur.edu.pl; 5 Medical Faculty, University of Rzeszów, 35-959 Rzeszów, Poland

**Keywords:** Alexander disease, spastic paraplegia, *GFAP* gene, late onset

## Abstract

**Objectives**: Alexander disease (AxD) is a rare neurodegenerative disorder that represents a group of leukodystrophies with severe disability and premature death, mostly with an infancy/childhood onset. In rare cases of late-onset phenotypes, symptoms are often milder and difficult to diagnose. We present a diagnostic journey of a teenage male patient with a progressive gait disorder starting at the age of 13 years, with a final diagnosis of Alexander disease. Early in the course of the disease, the boy exhibited distinctive cognitive involvement and neuropsychological deterioration characterized by selective impairment of visual and long-term auditory memory, along with a decline in IQ but preserved reasoning abilities. **Methods**: The patient underwent an extensive neurological diagnostic workup, which included magnetic resonance imaging (MRI) of the brain, spine, and abdomen, as well as electrophysiological, metabolic, and biochemical tests. Numerous specialist consultations were conducted, including genetic, cardiology, ophthalmology, pulmonology, oncohematology, psychological, and speech–language pathology consultations. In addition, a focused literature review was performed using PubMed, Scopus, Web of Science, and Google Scholar with the search terms “Alexander disease,” “*GFAP* gene,” “late-onset,” “spastic paraplegia” and “*GFAP variant p/Gly18Val*”. **Results**: Whole exome sequencing revealed an extremely rare missense GFAP heterozygous variant NM_002055.5: c.54G>T (*p/Gly18Val*), confirming the diagnosis of AxD. **Conclusions**: The presented case highlights the importance of whole-exome sequencing in the diagnosis of unexplained otherwise neurological symptoms, such as progressive spastic paraplegia.

## 1. Introduction

Alexander disease (AxD) is a rare leukodystrophy caused by gain-of-function mutations in the glial fibrillary acidic protein (GFAP) gene, located on chromosome 17q21, which encodes an intermediate filament protein primarily expressed in astrocytes [1]. Alexander disease has an autosomal dominant inheritance pattern; most cases result from a de novo mutation [2]. According to a Japanese population-based study, the prevalence is estimated at 1 in 2.7 million [3]. The GFAP variants exert gain-of-function effects, disrupting astrocytic intermediate filaments and producing cytoplasmic Rosenthal fibers—aggregates of GFAP, HSP27, and αB-crystallin that are pathognomonic [4].

Beyond protein aggregation, GFAP mutations appear to impair proteasomal activity and induce chemokine and nitric-oxide production, oxidative and cellular stress responses, astrocytic morphologic remodeling, and a pro-inflammatory CNS milieu; injury originates in astrocytes and likely propagates via microglial activation, culminating in white-matter degeneration and neuronal loss [5].

Clinically, AxD spans neonatal, infantile, juvenile, and adult forms [6] the neonatal form follows a rapidly progressive course with developmental delay/regression, seizures, and gastrointestinal symptoms [6,7,8]; the infantile form features developmental delay, seizures, ataxia, hyperreflexia/spasticity, hydrocephalus, and megalocephaly [9] the juvenile form includes milder delay with bulbar signs, vomiting, scoliosis, autonomic dysfunction, spasticity, ataxia, and epilepsy; and adult disease presents with bulbar or pseudobulbar features (e.g., palatal myoclonus, dysphagia, dysphonia, dysarthria), pyramidal gait disturbance, and cerebellar signs [10].

In 2001, Van der Knaap et al. identified, in infantile AxD, five magnetic resonance imaging (MRI) criteria: frontal-predominant cerebral white-matter abnormalities, a periventricular rim (T1 hyperintense/T2 hypointense), basal ganglia and thalamic involvement (swelling or atrophy), brainstem abnormalities (especially medulla and midbrain), and contrast enhancement of one of the following: the ventricular lining, periventricular rim, frontal white matter, optic chiasm, fornix, basal ganglia, thalamus, dentate nuclei, and brainstem [11].

Age-related phenotypes align into two radioclinical patterns. Type I AxD typically presents early with seizures, encephalopathy, episodic decompensation, failure to thrive, developmental delay, and a characteristic MRI pattern [12]. In contrast, type II AxD can manifest at any age and is characterized by autonomic and bulbar dysfunction, accompanied by ocular motor abnormalities and palatal myoclonus, while cognition and development are generally preserved. Emerging data indicate that the juvenile and adult subtypes are phenotypically indistinguishable and fit within the type II AxD spectrum [13,14]. At present, there is no biochemical marker for Alexander disease.

We present a case of a 14½-year-old boy with progressive spastic paraparesis with depressive symptoms and mild scoliosis as the only additional findings. This case should raise awareness for this rare and under-recognized disease in patients with progressive spastic paraparesis.

## 2. Materials and Methods

MRI (magnetic resonance imaging) of the brain was conducted on a GEMS Artist 1.5T system (GE Healthcare, Chicago, IL, USA) using the following sequences: SE (T1-axial), frFSE (T2-axial, coronal, sagittal), GRE (T2*-axial), IRFSE/FLAIRprop. (T2-axial), and SE/EPI (DWI/ADC-axial). Post-contrast imaging was performed with 3DBRAVO (T1-sagittal, 1.6 mm) and SE (T1-coronal, axial) sequences.

Whole-exome sequencing (WES) was performed, followed by an analysis of the identified variants. Particular attention was given to variants detected in genes listed in the OMIM database, in which defects are associated with the patient’s clinical manifestations. Additionally, the entire exome was analyzed for the presence of known pathogenic variants as well as rare, potentially pathogenic variants in genes whose defects are linked to the observed symptoms. WES was conducted on the proband’s DNA extracted from peripheral blood using Twist Human Core Exome Plus Kit Twist mtDNA Panel (Twist Bioscience, South San Francisco, CA, USA) according to the manufacturer’s instructions. The enriched library was paired-end sequenced (2 × 100 bp) on NovaSeq 6000 (Illumina, San Diego, CA, USA) (100× depth of mean coverage). The sensitivity of the method was confirmed, with total coverage at ≥30× depth exceeding 99%.

In addition, a focused literature review was performed using PubMed, Scopus, Web of Science, and Google Scholar databases, with search terms including “Alexander disease,” “*GFAP* gene,” “late-onset,” and “spastic paraplegia,” “*GFAP variant p/Gly18Val*,” to contextualize the clinical presentation and diagnostic findings.

## 3. Results

### 3.1. Case Presentation

A previously healthy 14½-year-old boy was admitted to the Pediatric Neurology Department with a progressive gait disturbance, beginning at age 13 with insidious lower-limb stiffness and weakness and in-toeing, more pronounced on the right, unresponsive to several months of prescribed physiotherapy after orthopedic evaluation. He had been a highly active cyclist, trained in jumping events, participated in school running competitions, and was a member of a local dance team. He was evaluated by an orthopedic surgeon and underwent a course of physiotherapy, but without improvement. Ongoing progression led him to withdraw from sports and dance.

The boy was born at 41 weeks after an uncomplicated pregnancy and delivery, with an Apgar score of 10 and a birth weight of 3800 g. Development was appropriate; he walked independently at 13 months. Past history is otherwise unremarkable, apart from recurrent streptococcal infections in early childhood (resolved by age eight) and surgical excision of a suppurated right lateral cervical (neck) cyst at age 12. Family history was available only on the maternal side and was negative for neurological disease.

Neurological examination revealed a head circumference of 54 cm (10th percentile for age) [15] with asymmetric cranial vaulting. Craniofacial and somatic dysmorphism included triangular facies, a short webbed neck, low posterior hairline, chest wall deformity, and a right supernumerary nipple; a postoperative scar was present along the right lateral neck, with scoliotic posture and mildly reduced cervical range of motion. Pupils were equal and reactive; extraocular movements were full without diplopia or nystagmus. Muscle tone was increased in the lower extremities; deep tendon reflexes were brisk in the upper and brisk with polyclonus in the lower extremities (right > left), with bilateral sustained ankle clonus (right > left) and bilateral extensor plantar responses (Babinski positive) (Video S1). Sensation to superficial and deep modalities was intact; coordination was preserved; the Romberg test was negative. Gait was paraparetic and inefficient, with bilateral foot drop and adducted foot posture (Video S2). Toe-walking was difficult, and heel-walking was not possible. He was able to squat and maintain a single-leg stance on the left but not on the right (Video S3). Multiple small ecchymoses were noted over the thighs and legs bilaterally, with mild distal edema of the lower limbs (right > left). The Body Mass Index (BMI) was 16.5 (3–10 percentile for age) [16].

**Figure 1:**
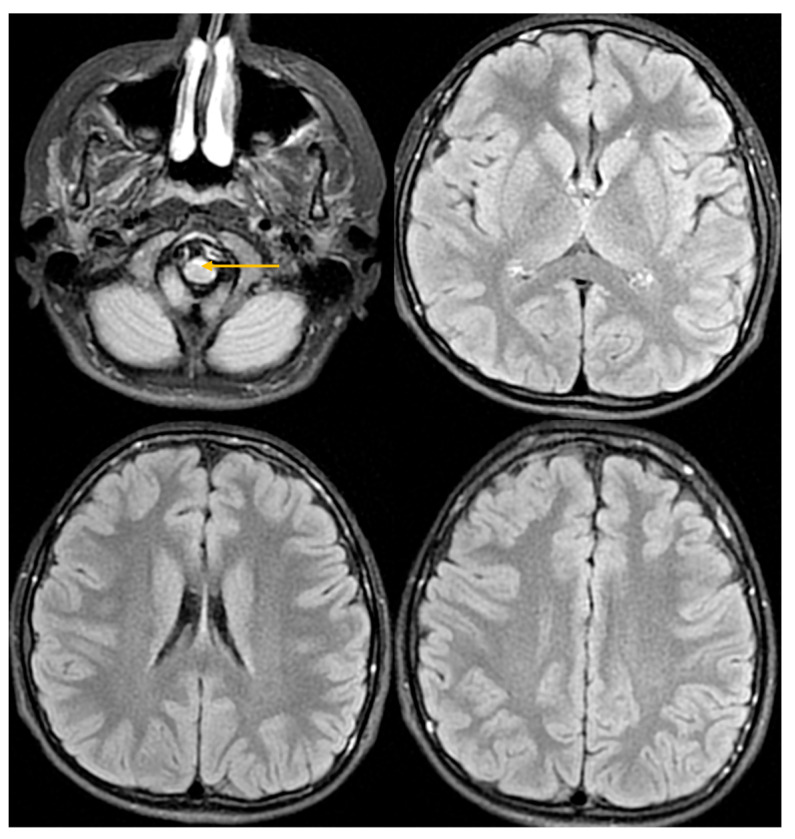
Brain MRI 11 April 2024. FLAIR sequence in axial projection. The arrow indicates an area of increased signal in the anterior part of the medulla oblongata.

A comprehensive imaging and laboratory workup was performed. Contrast-enhanced brain MRI was interpreted as otherwise normal, except for two pineal cysts measuring 2 × 6.5 × 2.5 cm and 6.5 × 10 × 6 cm (Figure 1). The radiological evaluation was changed at follow-up.

**Figure 2:**
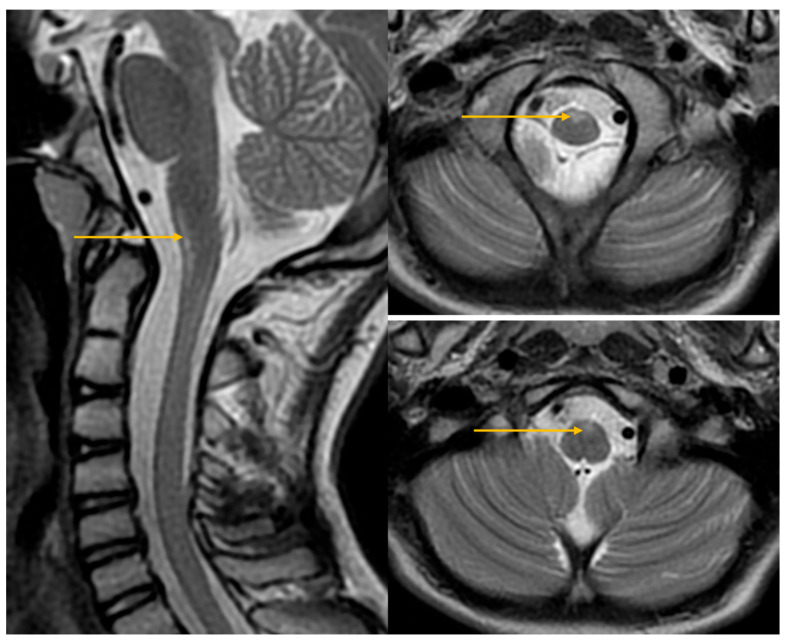
MRI of the cervical spine 11 April 2024FSE T2-weighted images in sagittal and axial planes. Arrows indicate areas of increased signal in the anterior portions of the medulla oblongata and the anterior horns of the spinal cord.

The MRI first report of the cervical spine contained no abnormal findings, which were changed during re-evaluation of the images during follow-up (Figure 2).

The thoracic spine MRI was normal except for a slight right-convex scoliosis. Contrast-enhanced abdominal/pelvic MRI was normal aside from a small right rectovesical fluid collection; no pathologic lymphadenopathy or muscle abnormalities. NCS (bilateral deep peroneal, tibial, ulnar) and EMG (right tibialis anterior) were normal. Abdominal/testicular ultrasonography and cervical spine, chest, and hip radiographs were unremarkable; ophthalmologic examination and funduscopy were normal bilaterally; ECG/echocardiography showed a structurally and functionally normal heart. Lumbar puncture yielded clear CSF (2 cells/µL, protein 27.7 mg/dL, chloride 121 mmol/L, glucose 57 mg/dL); CSF Borrelia testing, CSF onconeural antibodies, oligoclonal bands, and the serum/CSF autoimmune encephalitis panel were negative. Extensive laboratory studies showed lymphopenia, low-normal vitamin B12, vitamin D insufficiency, mild hyperhomocysteinemia, and mildly reduced IgA with normal IgG; tTG-IgA and tTG-IgG were negative; FSH and ceruloplasmin were within normal limits; HLA-B27 was not detected on microarray; rheumatoid factor, anti-GAD antibodies, and ANA-1 were negative; screening for HIV, Mycobacterium tuberculosis, Borrelia, and fecal calprotectin were negative. Speech–language evaluation revealed no speech or swallowing disorder. Psychological assessment showed average cognitive development; by history, he performed well academically with appropriate peer relationships. STAIC and CES-DC screening indicated elevated emotional tension with anxiety and depressive symptoms, without suicidal ideation.

The patient was referred to the Rehabilitation Department while awaiting results of an NGS panel for hereditary spastic paraplegia, a dried-blood-spot (DBS) assay for metachromatic leukodystrophy, and very-long-chain fatty acids (VLCFA) to exclude adrenoleukodystrophy. All these investigations were negative.

During subsequent hospitalizations three and four months later, neurologic examination showed further progression of the gait disorder; the patient could no longer toe- or heel-walk. The consulting oncohematologist advised serum neuron-specific enolase (NSE) and a 24 h urine collection for catecholamines and their acidic metabolites—both were within normal limits. The workup was extended to plasma and urine amino-acid profiles, which were normal. On repeated psychological evaluation, the Benton Visual Retention Test showed a sten score of 3 (6 errors; 6 correct), indicating mild visual memory impairment. IDS-2 testing demonstrated average intellectual functioning (IQ 98). Short- and long-term auditory memory scores were below average. The clinical geneticist recommended whole-exome sequencing (WES), and samples were obtained.

**Figure 3:**
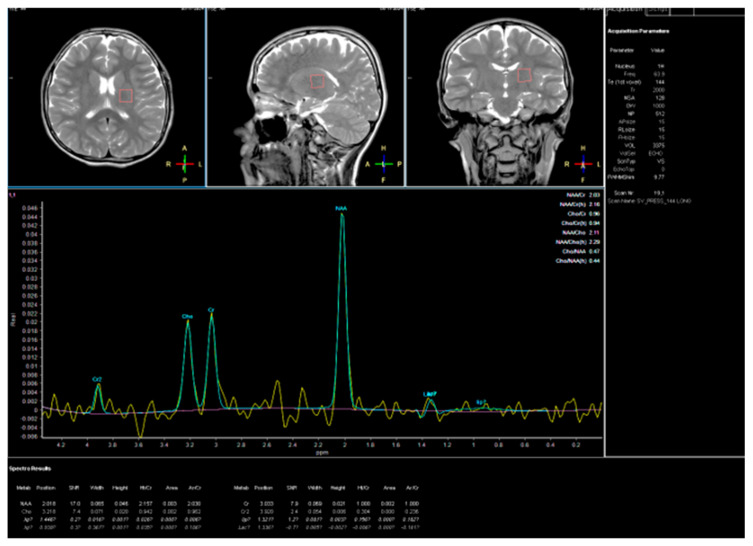
MRS examination 9 November 2024. H1-MR spectroscopy—normal image.

At age 15, seven months after the initial hospitalization, he was readmitted for reassessment after another 3 weeks of intense rehabilitation. Ankle Foot Orthosis (AFO) were prescribed for standing and walking. Neurologic examination documented further gait decline with right-predominant spasticity; he had difficulty lifting his legs and donning socks. In the lower extremities, deep tendon reflexes were polyclonic, with bilateral ankle clonus and right-sided patellar clonus. The boy required crutches for ambulation outside his room and reported intermittent abdominal paresthesias, particularly with exertion (e.g., walking). No additional symptoms were reported. Rehabilitation and pharmacological trials with sertraline, levodopa/benserazide, and baclofen yielded no observable clinical benefit, as reported by caregivers. A follow-up brain MRI with concurrent proton MR spectroscopy (^1^H-MRS) was performed. Multivoxel spectra acquired from frontal gray/white matter, deep nuclei, and the pre- and postcentral cortex showed normal metabolite profiles without abnormal ratios, providing no evidence of a neurodegenerative process (Figure 3).

Conventional MRI was unchanged from prior. Susceptibility-weighted and perfusion (PWI) sequences were unremarkable, and—together with ^1^H-MRS—did not demonstrate features typical of neurodegeneration. The whole exome sequencing (WES) evidenced a rare heterozygous missense variant: c.54G>T of the glial fibrillary acidic protein (GFAP) gene (*NM_002055.5*), which causes the glycine to valine amino acid substitution at codon 18 (*p/Gly18Val*) in the GFAP. This mutation has been reported only once in the literature in a familial case [17].

Given an unremarkable family history, the absence of abnormalities on brain and cervical MRI and MR spectroscopy, and the lack of characteristic clinical features of type II AxD—bulbar symptoms (dysarthria, dysphagia, dysphonia, palatal myoclonus), evident cerebellar ataxia, and nystagmus, this diagnosis was not suspected.

In the absence of a causal treatment, the patient was managed under the supervision of a physiotherapist. He underwent biweekly home-based rehabilitation sessions, performed prescribed exercises with parental assistance, and twice participated in intensive three-week inpatient rehabilitation programs, during which botulinum toxin was administered. Despite these comprehensive interventions, a progressive deterioration of gait was observed.

**Figure 4:**
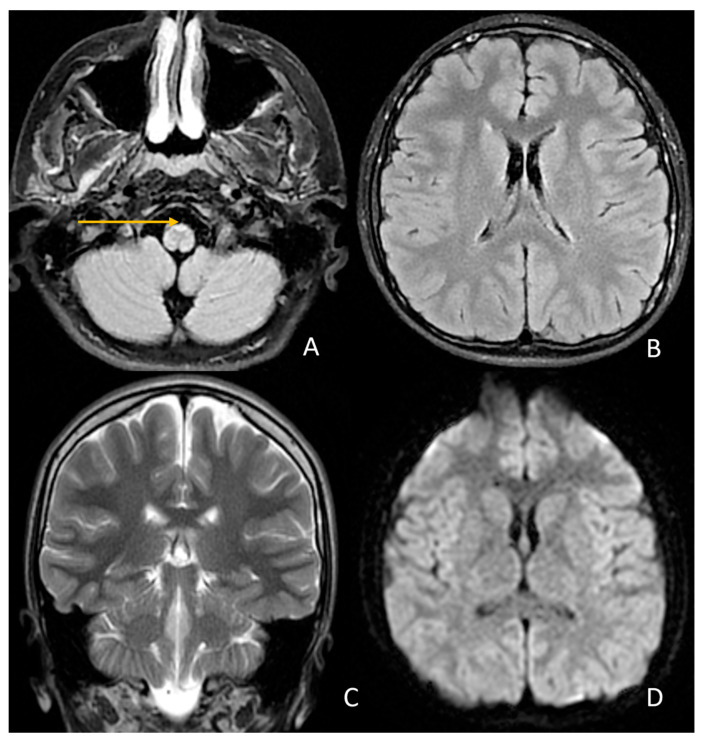
Follow-up MRI from 11 September 2025—stable image. (**A**)—FLAIR sequence—persistent band of increased signal in the anterior part of the medulla oblongata. (**B**)—FLAIR/T2 sequence in axial projection. (**C**)—FSE/T2 sequence in coronal projection. (**D**)—DWI sequence. (**B**–**D**)—normal image.

Sixteen months after the first hospitalization, the boy was admitted to the Department of Neurology and Pediatrics for follow-up imaging studies. Neurological assessment revealed progressive gait deterioration; the patient requires crutches or a wheelchair outside the home and uses environmental support for balance indoors (Videos S4 and S5). With the genetic diagnosis established, both the new and previous MRI scans of the brain and cervical spine were re-evaluated. Signal abnormalities were identified at the level of the lower medulla oblongata (between the pyramids) and in the anterior portion of the cervical spinal cord, visible on both axial and sagittal T2-weighted images (Figure 4).

**Figure 5:**
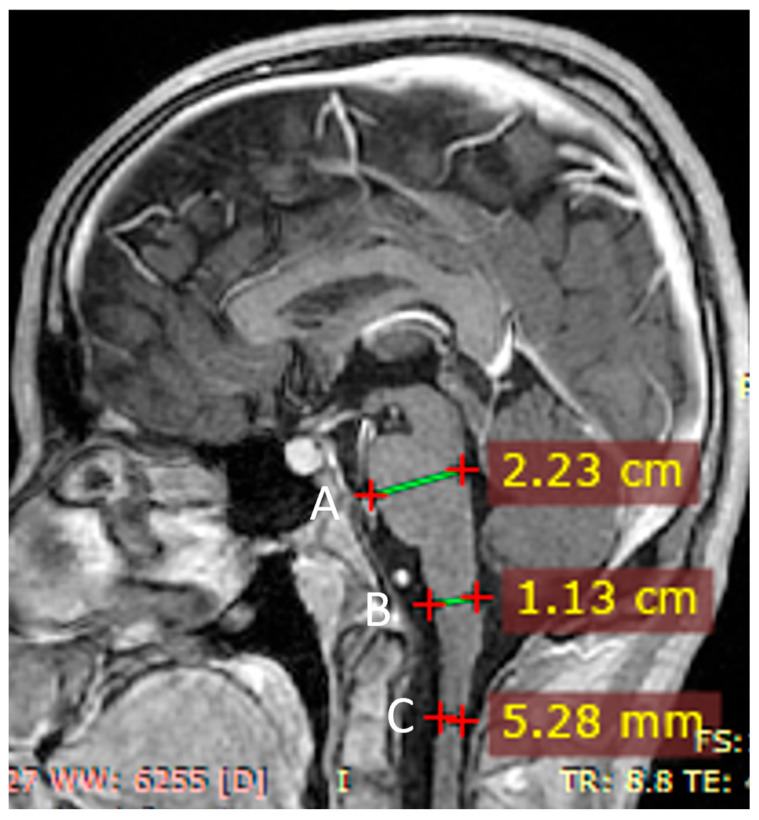
MRI examination—SE T1-weighted images after contrast administration 9 November 2024. B/A ratio (medulla oblongata/pons)—0.51. C—spinal cord diameter at the C2 level.

Persistent segmental spinal cord narrowing at C2 (sagittal diameter ~5.3 mm) was noted, which in previous examinations had been considered a constitutional feature. The width of the cervical spinal cord did not decrease in subsequent examinations (Figure 5).

Post-supplementation, previously low serum levels of vitamin B12 and vitamin D3, as well as homocysteine, were within normal reference ranges. However, neuropsychological assessment revealed a decline in intellectual functioning, with IQ decreasing from 98 to 85, though still within the average range; long-term auditory memory remained impaired. Emotional tension was present in both assessments, with predominant anxiety and depressive features noted initially, and a persistently low mood observed in the follow-up evaluations. Social functioning was previously adequate, but with difficulties in socially demanding situations. The consulting psychologist concluded that persistent emotional symptoms may adversely affect future social interactions. A comparative summary of the psychological assessments of our patient at the time of diagnosis and at follow-up is presented in (Table 1).

### 3.2. Literature Review

A focused literature review was conducted to summarize previously reported clinical, radiological, and genetic findings in adult-onset Alexander disease. The following section presents the most relevant data from published case reports and cohort studies, emphasizing features comparable to those observed in the present case.

Clinical spectrum and diagnostic criteria

Adult-onset Alexander disease (AxD) is the late-onset form of GFAP-related leukodystrophy, usually manifesting after adolescence with progressive spastic paraparesis and variable bulbar or cerebellar features [3]. In a nationwide Japanese survey, Yoshida et al. (2011) defined diagnostic criteria requiring onset after 12 years and at least one neurological and one radiological feature involving the medulla oblongata or cervical spinal cord [3]. Asymmetry of symptoms occurred in 35%, and dementia or rigidity in about 25–29% [3].

Genetic findings

Knuutinen et al. (2018) found GFAP variants mainly within exons 1–6, associated with pyramidal and bulbar signs and characteristic brainstem MRI changes [8]. Casasnovas et al. identified a heterozygous GFAP c.53G>T (*p/Gly18Val*) variant in six adults with slowly progressive spastic paraparesis, mild cerebellar signs, and scoliosis [7]. MRI revealed T2 hyperintensity of the medulla without supratentorial lesions [7]; the same variant was detected in the present case [7].

Neuroimaging characteristics

Van der Knaap et al. established MRI criteria showing T2 hyperintensity and atrophy of the medulla oblongata and upper cervical cord [11]. Yoshida et al. (2017, 2020, 2021) demonstrated that a medulla diameter < 9 mm and a medulla-to-pons ratio < 0.46 are typical for adult-onset AxD, with lesions confined mainly to the medulla [18,19,20]. Casasnovas et al. confirmed similar findings in *p/Gly18Val* carriers [7]. Peer et al. described a distinctive “frog-face” and “strangulated medulla” appearance linked to another GFAP mutation [15].

Clinical manifestations and disease course

Romano et al. reported progressive bulbar and pyramidal signs with medullary and cervical cord atrophy [9]. Yoshida et al. (2021) noted frequent bulbar and autonomic dysfunction in older-onset disease [20]. Sundblom et al. provided comparative MRI data for leukodystrophies with autonomic features [21].

Cognitive and experimental data

Kirsch et al. described attention and memory deficits in pediatric AxD [16], while Berman et al. showed in a GFAP mutant rat that astrocytic dysfunction leads to impaired synaptic plasticity and cognitive decline [17].

Across studies, adult-onset AxD associated with GFAP mutations—including *p/Gly18Val*—is defined by slowly progressive spastic paraparesis, mild bulbar or cerebellar signs, and restricted medullary MRI changes with preserved supratentorial structures [6,7,8,9,10,11,12,13,14,15]. These findings confirm phenotype variability and support GFAP sequencing and focused brainstem imaging in diagnosis [6,7,8,9,10,11,12,13,14,15].

## 4. Discussion

In late-onset Alexander disease, two subtypes are distinguished: the juvenile form, with symptom onset between 2 and 12 years of age, and the adult form, which begins in adolescence or adulthood [2,11].

Diagnostic criteria for adult-onset Alexander disease were defined by symptom onset after the age of 12 years and the presence of at least one clinical feature from group (a) and one radiological feature from group (b): (a) Neurological findings such as paralysis, bulbar or pseudobulbar signs, cerebellar ataxia, autonomic dysfunction, nystagmus, palatal myoclonus, or dementia. (b) MRI findings showing signal abnormalities or atrophy of the medulla oblongata and/or cervical spinal cord [3].

Our patient developed progressive spastic paraparesis as the sole major initial clinical manifestation, with symptom onset at the age of 13. Despite an extensive diagnostic workup, including brain and cervical MRI, the subtle radiological changes were initially interpreted as normal. The challenge of recognizing such findings has been highlighted previously [17], where similar difficulties were reported in the diagnostic process of the first identified family member with adult-onset spastic paraplegia caused by a novel GFAP N-terminal head domain variant (*p/Gly18Val*)—the same mutation detected in our young patient. The authors of this study identified 6 family members with the same mutation. They suggested, based on clinical data and functional studies, that this variant is less deleterious than the vast majority of Alexander disease mutations, giving rise to an attenuated clinical phenotype. To our knowledge, our patient is the second reported unrelated case carrying this rare variant. Unfortunately, genetic testing of the patient’s family for the presence of the pathogenic variant could not be performed. The family history, to the extent it could be obtained, was negative for clinical features of Alexander disease. In Table 2 we compared the clinical findings in our patient with those reported by Casasnovas et al. [17].

Our patient exhibited more pronounced pyramidal symptoms and muscle rigidity in the right limb. No information regarding symptom asymmetry was provided for the analyzed group of patients, however in a nationwide survey of Alexander disease conducted in Japan, asymmetry was observed in at least 35% of adult cases. Additionally, dementia and muscle rigidity were reported in 25% and 29.4% of patients, respectively [3]. Our patient did not exhibit the oculomotor abnormalities reported in all other individuals carrying the same mutation, nor the bladder dysfunction described in two of the six familial cases. We believe that this discrepancy may be related to the relatively short interval between symptom onset and diagnosis in our patient (approximately two years), compared with the longer diagnostic delays of three to thirteen years reported in other cases [17]. It is therefore plausible that these additional symptoms may develop over time as part of the natural course of the disease. This is a limitation of the short follow-up period in our case and underlines the importance of longitudinal monitoring to capture the full phenotypic spectrum associated with this mutation. It is noteworthy that neither the familial case reported by Casasnovas et al. [17] nor our patient carrying the *p/Gly18Val* mutation in the *GFAP gene* exhibited bulbar symptoms such as dysphagia, dysarthria, or palatal myoclonus, which are typical for AOAD [3], nor did they present with features of dysautonomia. This consistent absence of bulbar and autonomic involvement may indicate a distinct phenotypic profile associated with this particular variant and raises the possibility of a milder disease course, although further cases are needed to confirm this observation. No information regarding intellectual decline was available for the reported six patients with *p/Gly18Val* mutation in the GFAP gene [17]. Current literature provides limited evidence on the extent of neurocognitive decline in patients with disease onset during early adolescence and on the prognosis for subsequent developmental outcomes. Yoshida et al. reported dementia in 25% of the adult-onset cohort; however, there were no adolescent patients in the group [3]. Another case report highlighted intellectual decline in a teenage patient with type II Alexander disease [21]. In a recent study, Berman et al. demonstrated that Gfap^+^/R237H rats display impaired synaptic plasticity and cognitive deficits as additional clinically relevant phenotypes. Transcriptomic analysis of the hippocampus in young adult animals revealed a neurodegenerative profile characterized by activation of the innate immune response and downregulation of synaptic and metabolic genes which are features commonly associated with chronic neurodegenerative disorders of aging [22]. In our patient, we observed a decrease in IQ from 98 to 85 at the 16-month follow-up. The neuropsychological profile was characterized by selective impairment of visual and long-term auditory memory, with preserved reasoning abilities. This pattern, together with the observed IQ decline, may indicate early cognitive involvement and neuropsychological deterioration. It is noteworthy that there was a discrepancy between the patient’s self-reported “fairly good” school functioning during the psychological follow up assessment and the school’s report, which suggested otherwise: “The boy adopts a passive attitude during lessons. He was at risk of failing Polish, German, and Mathematics, but by the end of the first semester, he had no failing grades, obtaining minimum passing grades in about half of the subjects. He also shows difficulties with attention and, at times, in the socio-emotional domain”.

In Table 3 we compared the radiological features of AOAD in our patient and those reported by Casasnovas et al. [17] with the rare *GFAP* variant (*p/Gly18Val*) using the diagnostic criteria proposed by van der Knaap et al. and Yoshida et al. [11,18,23,24,25].

Yoshida et al. reported that a sagittal diameter of the medulla oblongata below 9.0 mm and a sagittal MO/Po ratio below 0.46 demonstrated high sensitivity and specificity for AxD [19]. In another study in older adults with bulbospinal AOAD the mean diameter of the medulla oblongata was 7.7 mm and ratio of the medulla to pons was 0.36 mm [20]. In a recent scientific communication [26] the authors derived the pons/medulla oblongata ratio in 10 MRI scans of patients with no abnormality in the brain- stem and the average ratio was found to be 0.59, while the medulla oblongata to pons ratio in the reported AxD case was 0.35. To assess medulla oblongata atrophy, we measured the medulla oblongata-to-pons ratio, which was 0.51. Based on this finding, we concluded that there was no significant medullary atrophy in our patient. At the C2 level, the spinal cord’s anterior–posterior diameter was 5.28 mm, significantly below the established normative value of 9.5 ± 0.6 mm [27]. Despite minimal progression of spinal cord atrophy, our patient showed marked gait deterioration over the 16 months between the first and last follow-up assessments. To date, no studies have characterized the rate of spinal cord atrophy progression over time in relation to the clinical course of the disease.

## 5. Conclusions

This case of a patient with an extremely rare missense GFAP heterozygous variant NM_002055.5: c.54G>T (*p/Gly18Val*) illustrates that isolated spastic paraparesis in an adolescent should also prompt diagnostic evaluation for rare leukodystrophies. MRI assessment should include careful analysis for sometimes overlooked subtle findings, such as spinal cord narrowing or faint signal abnormalities within the medulla oblongata. With Zilganersen as the first potential treatment option for this disease emerging in the near future [28], early and accurate diagnosis becomes increasingly important.

## Figures and Tables

**Table 1:** Comparative Summary of Psychological Assessments.

Domain	July 2024 (Age 14 Year 10 Month	September 2025 (Age 16 Year)	Change/Observation
Contact, Orientation, Affect	Normal contact and orientation; affect appropriate; patient tense	Normal contact and orientation; affect appropriate; emotional tension and sadness noted	Persistent emotional tension, increased sadness
School and Social Functioning	Grade 8 student, learning fairly well; positive peer relations; anxiety in social situations (somatization)	Limited data; emotional tension and sadness possibly affecting functioning	Possible impact of emotional state on functioning
Emotional Assessment	STAIC and CES-DC: anxiety and depressive symptoms (no suicidal thoughts)	Observation: emotional tension, sad mood (patient denies low mood)	Persistent affective symptoms
Visual Memory (Benton)	Impaired (sten 3, 6 errors; population mean 6)	No data available	—
Short-term Auditory Memory (IDS-2)	WP = 6 (below average)	WP = 7 (average)	Improvement
Long-term Auditory Memory (IDS-2)	WP = 6 (below average)	WP = 6 (low)	No change
Abstract Reasoning (Matrices)	WP = 9 (average)	WP = 7 (average)	Slight decline
Conceptual Reasoning (Categories)	WP = 10 (average)	WP = 8 (average)	Slight decline
Intelligence Quotient (IDS-2)	IQ = 98 (average)	IQ = 85 (lower end of average)	Decrease in overall cognitive performance

**Table 2:** Clinical findings in patients with heterozygous missense variant in the *GFAP* gene, *p/Gly18Val*. F: Female, M: Male.

Symptom/Feature	Our Patient	Patient I:2	Patient II:2	Patient II:3	Patient II:5	Patient III:4	Patient III:5
Sex	M	M	F	F	F	M	M
Age at onset	13	NA	NA	46	17	10	9
Age of diagnosis	15	73	49	50	40	40	16
Spastic paraparesis	Yes	No	No	Yes	Yes	Yes	Yes
Pyramidal signs	Yes	Yes	Yes	Yes	Yes	Yes	Yes
Ataxia	Mild	No	No	Mild	Mild	Mild	Mild
Bulbar symptoms	No	Subtle	Subtle	Yes	Yes	Yes	Yes
Gait abnormalities	Yes	Yes	Yes	Yes	Yes	Yes	Yes
Scoliosis	Yes	No	Yes	Yes	No	Yes	Yes
Neurocognitive deficits	Yes	No	No	No	No	No	No
Ocular movement abnormalities	No	Not reported	Yes (nystagmus, diplopia)	Yes (nystagmus)	Yes	Yes	Yes
Dysarthia/dysphagia	No	No	No	No	No	No	No
Bladder dysfunction	No	No	No	Yes	Yes	No	No
Palatal tremor	No	No	No	No	No	No	No
Dysautonomia	No	No	No	No	No	No	No
References	this paper	[17]					

**Table 3:** Radiological findings.

Symptom/Feature	Our Patient	Patient I:2	Patient II:2	Patient II:3	Patient II:5	Patient III:4	Patient III:5
Abnormal signal intensity of the anterior portion of the medulla oblongata	Yes	NA	Yes	Yes	Yes	Yes	Yes
Atrophy of the medulla	No	NA	No	Yes	Yes	Yes	Yes
Atrophy of the cervical spinal cord	Yes	NA	No	Yes	Yes	Yes	Yes
Signal abnormalities in the cerebellar white matter or hilus of the dentate nucleus	No	NA	No	No	No	No	No
Cyst formation in white matter around the anterior horn of the lateral ventricles	No	NA	No	No	No	No	No
Ventricular garlands	No	NA	NA	NA	NA	NA	NA
References	This paper	[17]					

## Data Availability

The original contributions presented in the study are included in the article and Supplementary Materials; further inquiries can be directed to the corresponding author.

## Supplementary Materials

The following supporting information can be downloaded at https://www.mdpi.com/article/10.3390/jcm14228232/s1: Video S1: Tendon reflexes at baseline; Video S2: Gait at baseline; Video S3: One-leg stance and crouching at baseline; Video S4: Gait at follow-up; Video S5: Standing-up at follow-up.
